# Help-seeking during 1-year follow-up in Chinese patients diagnosed with type 2 diabetes mellitus comorbid major depressive disorder

**DOI:** 10.3389/fendo.2023.1266183

**Published:** 2023-10-10

**Authors:** Wenqi Geng, Yinan Jiang, Xia Hong, Weigang Zhao, Jie Ren, Cathy Lloyd, Norman Sartorius, Jing Wei

**Affiliations:** ^1^ Department of Psychological Medicine, Chinese Academy of Medical Sciences & Peking Union Medical College, Peking Union Medical College Hospital, Beijing, China; ^2^ Department of Endocrinology, Key Laboratory of Endocrinology of National Health Commission, Chinese Academy of Medical Sciences & Peking Union Medical College, Peking Union Medical College Hospital, Beijing, China; ^3^ Department of Psychiatry, Beijing Xicheng District Pingan Hospital, Beijing, China; ^4^ Faculty of Wellbeing, Education and Language Studies, The Open University, Milton Keynes, United Kingdom; ^5^ Association for the Improvement of Mental Health Programmes (AMH), Geneva, Switzerland

**Keywords:** type 2 diabetes mellitus, major depressive disorder, comorbidity, cohort study, help seeking

## Abstract

**Introduction:**

Previous research has revealed a bidirectional relationship between type 2 diabetes mellitus (T2DM) and major depressive disorder (MDD). A very limited proportion of patients with T2DM comorbid MDD received adequate psychiatric intervention. This study investigated the help-seeking behaviors of patients with T2DM comorbid with MDD during one-year follow-up.

**Methods:**

At a medical center in China, a cohort of outpatients with T2DM were assessed and diagnosed for comorbid depression at baseline and after one year. The Mini International Neuropsychiatric Interview was used to diagnose MDD, while The Patient Health Questionnaire-9 (PHQ-9) and The Hamilton Depression Scale 17-item (HAMD-17) were used for depression assessment. Mental health help-seeking behaviors of patients during follow-up period were also evaluated.

**Results:**

Out of the 203 patients with T2DM at baseline, 114 (56.2%) completed the follow-up. The prevalence of MDD in participants with T2DM was 12.8% at baseline and 22.8% at follow-up. Patients who completed the follow-up had a lower baseline PHQ-9 score (test statistic -2.068, p=0.039), HAMD-17 score (test statistic -2.285, p=0.022) than those who did not complete the follow-up. A total of 26 patients had comorbid MDD during the follow-up period, among which 8 patients (30.8%) voluntarily visited psychiatric clinics, while others did not seek assistance. The level of HbA1c at follow-up was higher in patients who sought help than in those who did not (8.1 ± 1.8% *vs.* 7.0 ± 0.7%), although the difference was not statistically significant.

**Conclusion:**

Voluntary psychiatric help-seeking for Chinese patients with comorbid T2DM and MDD is uncommon. It is crucial to increase awareness of depression among patients and healthcare professionals alike.

## 1 Introduction

Diabetes and depression are both among the top causes of disability-adjusted life-years in the adult population (1). Patients diagnosed with Type 2 Diabetes Mellitus (T2DM) require long-term medical care, self-management, family support, and health education to prevent or delay the development of complications (2). However, chronic physical diseases often cause psychological distress in patients, which in turn affects patients’ ability and motivation for disease management (3). The prevalence of major depressive disorder (MDD) in patients with T2DM was 7.4% in a multicenter study across 12 countries (4). Previous research has revealed a bidirectional relationship between diabetes and depression (5). Patients with T2DM have a higher prevalence of depression than the general population (6). Depression was found to be an independent risk factor for worse glycemic control and associated with a 1.7-2.0 fold higher prevalence of complications in diabetes (7). In a recent epidemiological study of mental disorders in the Chinese population, only 11.6% of patients with MDD actively sought any form of treatment and only 0.8% were considered to have received adequate treatment (8). There are few studies on psychiatric help-seeking in patients with T2DM. In this study, we focused on the help-seeking behaviors of patients with T2DM comorbid with MDD during 1-year follow-up.

## 2 Methods

### 2.1 Study design

This study was conducted as part of the International Prevalence and Treatment of Diabetes and Depression (INTERPRET-DD) study, a two-year international longitudinal study carried out across 16 countries including Argentina, Bangladesh, Brazil, China, Germany, India, Italy, Kenya, Mexico, Pakistan, Poland, Russia, Serbia, Thailand, Uganda, and Ukraine. A comprehensive protocol outlining the study design and procedure for data collection in the INTERPRET-DD study has been previously published (9). The primary goals of the initial research were to assess the prevalence and incidence (over 12 months) of depressive disorders and emotional distress related to diabetes in adults with T2DM, as well as to investigate how frequently depression is documented/undocumented in individuals with T2DM and examine which care pathways are implemented for depression. Our current study aims to investigate the help-seeking behaviors of patients with comorbid T2DM and MDD during a 1-year follow-up period.

As one of the centers participating in the INTERPRET-DD study, we conducted convenient sampling to recruit participants from July 2014 to March 2015 in the outpatient clinic of the Department of Endocrinology at Peking Union Medical College Hospital, which is a tertiary general hospital located in Beijing, China. Inclusion criteria were: (1) 21-65 years of age, (2) outpatients, (3) diagnosed with T2DM at least 12 months before enrolment. Exclusion criteria were: (1) diagnosed with T2DM less than 12 months before enrolment, (2) unable to complete survey tools due to communication or cognitive difficulties, (3) inpatients, (4) any life-threatening or serious conditions, (5) pregnant women or women who gave birth within the last 6 months.

This study was approved by the Ethics Committee of Peking Union Medical College Hospital. Written informed consent was obtained from each participant.

### 2.2 Data collection

Measurements of medical and psychological characteristics were performed at enrolment and after one year. Upon enrolment, participants completed a survey on sociodemographic information including age, sex, level of education, occupation, and marital status. The researchers extracted clinical data for each participant from the hospital’s electronic database including height, weight, level of HbAlc, course of disease, T2DM complications, family history, and medication plan. Psychological evaluation included self-rating questionnaires and structured clinical diagnostic interviews (see 2.3 Instruments). If a participant was diagnosed with MDD after the diagnostic interview, the researcher would inform the participant and suggest visiting the Department of Psychological Medicine in the hospital. One year after enrolment, participants were contacted and invited to revisit the Department of Endocrinology outpatient clinic and complete the follow-up survey. Patients diagnosed with MDD at baseline were also asked to share information on whether or not they had sought any kind of help with depression in the past year.

### 2.3 Instruments

#### 2.3.1 The Mini International Neuropsychiatric Interview

A structured diagnostic tool for mental disorders developed by Sheehan and Lecrubier, which has been widely used in a range of different populations (10). In the validation study, MINI had a positive predictive value of 84.3% for MDD in Chinese patients (11). MINI assessments were conducted by two expert psychiatrists, who were blind to the medical and psychological status of the participants.

#### 2.3.2 The Patient Health Questionnaire-9, PHQ-9

A 9-item self-rating screening scale for depressive symptoms. The presence of MDD was defined as PHQ-9 scores >9 in a validation study in Chinese outpatients (12).

#### 2.3.3 The Hamilton Depression Scale 17-item, HAMD-17

A 17-item examiner-rating scale for depressive symptoms. Items are divided into five dimensions: psychomotor retardation, cognitive impairment, anxiety or somatization, sleep symptoms, and weight change. HAMD-17 scores >17 indicate the presence of depressive symptoms (13). Two trained psychiatrists performed HAMD-17 for all participants.

#### 2.3.4 Problem Areas in Diabetes Scale, PAID

A 20-item self-rating scale compiled by Polonsky et al. in 1995, PAID involves questions about stress experienced by patients with diabetes. The Chinese version has four factors, emotional, therapeutic, dietary, and social support. A total score of >40 indicates disease stress with diabetes (14).

### 2.4 Statistical analysis

All statistical analyses were performed using IBM SPSS Statistics 21.0.0.0. (IBM Corp., Armonk, NY, USA). Quantitative variables are described as mean ± standard deviation or median (interquartile range) based on the normality of the variable. Categorical variables were described as frequencies (percentages). Student t-test and χ^2^ test were used to compare continuous and categorical variables between groups. The Mann-Whitney test was used to compare non-normalized variables between groups. A value of p < 0.05 was considered statistically significant.

## 3 Results

A total of 203 patients with T2DM were included at baseline (**Table 1**). The average age of the patients was 53.5 ± 9.8 years, with 95 (46.8%) female patients. The median disease course of T2DM was 8 (4, 14) years, and the average HbA1c was 7.47 ± 1.90%. Complications were prevalent, with 44 (21.7%) experiencing cardiac issues, 33 (16.3%) renal complications, 70 (34.5%) ocular complications, and 44 (21.7%) peripheral nerve complications. In terms of patient-reported outcomes, the median PAID scale score was 15 (6, 27) points, compared to a median PHQ-9 score of 4 (2, 8) and a median HAMD-17 score of 5 (2, 11). 26 (12.8%) patients had comorbidity of MDD.

**Table 1:** Comparisons of clinical characteristics of participants at baseline and follow-up.

At baseline	Participants who completed follow-up (n=114)	Participants who did not complete follow-up (n=89)	p	At follow-up	Participants with comorbid MDD and T2DM (n=17)	Participants without comorbid MDD and T2DM (n=97)	p
HbA1c(%)	7.4 ± 1.8	7.6 ± 2.0	>0.05	HbA1c(%)	7.3 ± 1.6	7.2 ± 1.2	>0.05
BMI (kg/m^2^)	25.6 ± 3.6	25.9 ± 3.6	>0.05	BMI (kg/m^2^)	25.6 ± 3.1	25.8 ± 3.6	>0.05
Hyperlipidemia	83(72.8)	69(77.5)	>0.05	Hyperlipidemia	11(64.7)	91(93.8)	<0.001
PHQ-9	3(1,8)	5(3,9)	0.039	PHQ-9	17(9,21)	3(1,5)	<0.001
HAMD-17	4(1,11)	7(3,12)	0.022	HAMD-17	23(19,29)	7(3,15)	<0.001
PAID	12(5,22)	19(7,31)	0.041	PAID	20(11,48)	7(2,15)	0.006
MDD	17(14.9%)	9(10.1%)	>0.05				

MDD, Major depressive disorder; T2DM, Type 2 diabetes mellitus; PHQ-9, The Patient Health Questionnaire-9; HAMD-17, The Hamilton Depression Scale 17-item; PAID, Problem Areas in Diabetes Scale. Quantitative variables are described as mean ± standard deviation or median (interquartile range).

At 1-year follow-up, a total of 114 patients (56.2%) completed the study, with an average age of 54.2 years. Of these patients, 60 (52.6%) were female, and the majority (96.5%) were married. 26 (22.8%) of them were comorbid with MDD. Nine patients were newly diagnosed with MDD. Six patients reported clinical remission from a major depressive episode during the follow-up period, thus not meeting the criteria of MDD at follow-up. The 1-year incidence rate of MDD in this study was 13.2% (15/114).

### 3.1 Follow-up comparisons

Patients who completed the follow-up had a lower baseline PHQ-9 score (test statistic -2.068, p=0.039), HAMD-17 score (test statistic -2.285, p=0.022), and PAID score (test statistic -2.047, p=0.041) than those who did not complete the follow-up. There were no significant differences in age, sex, marital status, occupation, level of education, body mass index, disease course, HbA1c, complications, and prevalence of MDD between the two groups.

Of the 26 patients diagnosed with MDD at baseline, 17 (65.4%) completed follow-up at 1 year. Differences between patients with or without comorbid MDD in HbA1c, complications, clinic visits, and frequency of hospitalization were not statistically significant. Patients with comorbid MDD at baseline had a higher PHQ-9 score (test statistic 4.164, p<0.001), PAID score (test statistic 2.728, p=0.006), and HAMD-17 score (test statistic 3.997, p<0.001) at follow-up than those without comorbidity.

### 3.2 Psychiatric help-seeking of patients with T2DM comorbid MDD

There were no significant differences in sociodemographic characteristics, scores of depressive symptoms, and scores of diabetes distress between patients who sought psychiatric help and patients who did not. The level of HbA1c at follow-up was higher in patients who sought help than in those who did not (8.1 ± 1.8% *vs.* 7.0 ± 0.7%), although the difference was not statistically significant.

Of the 17 patients with MDD at baseline who completed follow-up, five engaged in active self-help and did not meet the diagnosis of MDD at 1 year thus considered to be in remission of depression. Five patients visited psychiatric clinics after baseline assessment. However, they did not achieve remission at follow-up. Seven patients had never sought help for depression and gave different reasons (**Table 2**). Three of the nine patients newly diagnosed with MDD at follow-up had acquired psychiatric help from clinics. The other six were unaware of their comorbidities and did not seek help.

**Table 2:** Help-seeking of 26 patients with comorbid T2DM and MDD.

Participants who sought help (13)	Time of diagnosis	Description of help	Participants who did not seek help (13)	Time of diagnosis	Reasons for not having sought psychiatric help
2 (in remission)	Baseline	Changes in attitudes and coping strategies towards T2DM	2	Baseline	Prefer self-help
1 (in remission)	Baseline	Actively manage T2DM symptoms	2	Baseline	Do not acknowledge diagnosis of MDD
1 (in remission)	Baseline	Actively improve marital relationships	1	Baseline	Medical insurance does not cover mental health
1 (in remission)	Baseline	Antidepressants (self-prescribed)	1	Baseline	Difficult to get appointments
5	Baseline	Psychiatric clinic (antidepressants and/or psychotherapy)	1	Baseline	Unwilling to go to a psychiatric clinic
3	Follow-up	Psychiatric clinic (antidepressants and/or psychotherapy)	6	Follow-up	Not aware of MDD diagnosis

T2DM, Type 2 Diabetes Mellitus; MDD, Major Depressive Disorder.

## 4 Discussion

Depressive symptoms and disease distress are common in patients with T2DM (15–17). Previous studies have noted discrepancies between the prevalence of depressive symptoms and the diagnosis of MDD (18). Self-rating depression scores may be influenced by concomitant diabetes-specific distress (4, 18). Our study included both psychiatric diagnoses of MDD and self-rating depression scores, providing concrete information on the prevalence of comorbid MDD and T2DM. Help-seeking behavior reflects one’s attitude and awareness of the state of health, which in turn affects the treatment process and outcome of one’s disease (19). Therefore, it is necessary to investigate help-seeking behavior in patients with chronic diseases whose management requires continuous effort and good coherence. Few studies have specifically focused on those with T2DM and MDD comorbidity (16, 20). In our study, we also included the mental health help-seeking characteristics of patients with T2DM, hoping to provide more information on this topic.

It is noteworthy that the prevalence of MDD in participants with T2DM in our study at baseline (12.8%) and follow-up (22.8%) were both higher than the 12-month prevalence of MDD (3.9%) in the general Chinese population (8). Results from the INTERPRET-DD study in 12 countries (China not included) suggested that the incidence rate of MDD in T2DM was 7.4%, including both baseline and follow-up data of 1616 patients, the highest found in Ukraine (35.4%), Pakistan (10.8%), and India (9.5%) (4). In a multi-center cross-sectional study of 2538 Chinese outpatients of T2DM, 6.1% had depression, which was defined as a PHQ-9 score > 9 (7). The high prevalence of MDD in our study may stem from a relatively small sample, loss of follow-up in some patients, and differences in hospital settings.

Similar to most diseases, etiology of T2DM or MDD is complex, not simply “There’s something wrong with my body/mind,” but often involves multiple bio-psychosocial factors. The same is true of why patients do not seek help, not simply “I don’t want to go.” Only 19.2% of patients with comorbid MDD in our study accepted referral to a psychiatric clinic where they received treatment for depression. With the exception of patients who were not aware of their mental status being considered depressive and therefore did not visit psychiatric clinics, the rest majority showed reluctance to see a psychiatrist. Help-seeking in patients with MDD may be a good example to explain the bio-psychosocial factors. Biologically, diagnostic criteria for MDD include decreased physical strength and motivation, as well as impaired cognitive function, all of which may act as biological barriers to help-seeking behavior. Psychosocially, attribution, cognition and perception of disease, doctor-patient relationship, and coping strategies all affect one’s help-seeking behavior. Patients’ attribution and cognition of symptoms or diseases often affect their help-seeking behavior (21, 22). Some of the patients in our study attributed depression either to difficulties in T2DM management, i.e. illness distress, or psychosocial distress such as relationship problems. In the meantime, they also attributed the remission of depression to the elimination of the distress mentioned. Another factor is the stigma associated with the diagnosis of mental disorder, still present in China and many countries (22–25), which may explain to some extent why a patient in this study would rather self-administer antidepressants than obtain adequate intervention from psychiatric clinics. Some patients prefer to describe emotional feelings using terms such as “tension,” “stress,” instead of medical terms such as “depression” and “anxiety” (26). Others tend to express concerns about physical symptoms rather than emotional symptoms (27). These phenomena may affect the physician’s interpretation of the patient’s underlying condition. Certain physical symptoms, such as gastrointestinal symptoms, exist in both T2DM and MDD, making differential diagnosis an even more difficult task for physicians. In our study, the researchers directly recommended that participants visit psychiatric clinics upon diagnosis of MDD, which is different from clinical settings, where usually the treating physician would decide whether or not to refer a patient. Doctor-patient rapport plays an important role in patient coherence, and a good doctor-patient relationship usually promotes patient help-seeking behavior (19). It has been reported that in general hospitals in China, the recommended referral rate for patients with depression was only 19.4% (28), while in low- and middle-income countries, about 80-90% of patients with depression have not been diagnosed or treated (29). As shown in our study, if neither physicians nor patients themselves were aware of patients’ mental health condition, the idea of referral to the psychiatric department would not spontaneously occur in patients with T2DM. Given the significance of treating comorbid MDD (7), it is important to include routine screening and assessment of psychological and social status in patients with T2DM, as indicated in the American Diabetes Association (ADA) treatment guidelines (2). Coping strategies, or coping resources, refer to efforts to manage the demands created by stressful events, such as chronic disease, in which social support plays an important role (30). A meta-analytic review suggested that as individuals in Asia or with Asian origins may be affected by collectivistic cultural orientation, they tend to deal with difficulties first within a core unit such as family (31), rather than reaching out for other resources. It was also suggested that treatment-seeking was associated with perceived failure of coping strategies (21). Some participants in our study expressed a preference for self-help, which could be interpreted as the need for autonomy and self-esteem. It is also possible that they did not experience depression as a serious condition, as the severity of the illness was found to be a factor affecting help-seeking behavior (17, 19, 22). Sample size limits the interpretation of some results. For example, it is possible that patients with worse glycemic control would be more proactive in seeking medical advice, which includes referral to another department. Although the proactive pursuit of medical and psychiatric support typically indicates good coherence, which tends to facilitate better glycemic management, there is evidence to suggest otherwise, as our study has demonstrated. In our view, patients experiencing poorer glycemic control are likely to suffer a greater degree of physical and psychological distress, leading them to actively seek assistance more urgently.

Despite the limited sample size, our study observed a relatively high drop-out rate. Previous research has examined potential predictors of drop-out for diabetes patients in cohorts. One program for T2DM management in Germany spanning over two years saw only 5.5% of 10,989 participants discontinue their enrollment, attributed to numbers of assessed factors upon program entry (32). Another study found that initial high HbA1c levels were more indicative of eventual drop-out, with a rate of 13.2% among diabetes patients (33). However, our study did not observe significant differences in HbA1c levels between those who completed follow-up and those who dropped out, which suggests glycemic control may not be the primary contributor to drop-out. A fever surveillance cohort study suggests that participants’ background characteristics and perceptions can influence drop-out rates (34). Our study’s low psychiatric help-seeking rates and observation of lower depressive symptoms in those who dropped out suggest that our limited participant group may have been unwilling to continue with follow-up, regardless of their condition being diabetes or depression.

Our study has several strengths. The first is the use of diagnostic interviews to assess depression, which provides a more accurate prevalence of MDD in this group of patients with T2DM. Secondly, the prospective design of follow-up allows us to learn about patients’ help-seeking behaviors during the period between enrolment and follow-up. Third, we provided both qualitative and quantitative data on participants. However, the relatively small sample size, the study design of a single center, and the loss of follow-up of more than 40%, all limit the generalizability of our study.

## 5 Conclusions

In conclusion, the comorbidity of MDD in patients of T2DM is not uncommon in China, but few seek for psychiatric help. To provide adequate psychiatric intervention for patients with comorbidity, it is important to take a comprehensive multidisciplinary approach to raising awareness of depression among both patients and physicians.

## Data availability statement

The original contributions presented in the study are included in the article/supplementary material. Further inquiries can be directed to the corresponding authors.

## Ethics statement

The studies involving humans were approved by Ethics Committee of Peking Union Medical College Hospital. The studies were conducted in accordance with the local legislation and institutional requirements. The participants provided their written informed consent to participate in this study.

## Author contributions

WG: Conceptualization, Data curation, Writing – original draft, Writing – review & editing. YJ: Conceptualization, Data curation, Writing – original draft, Writing – review & editing. XH: Conceptualization, Supervision, Writing – review & editing. WZ: Supervision, Writing – review & editing. JR: Data curation, Writing – review & editing. CL: Supervision, Writing – review & editing. NS: Supervision, Writing – review & editing. JW: Conceptualization, Supervision, Writing – review & editing.
